# Perception of Biosimilar Biologics and Non-Medical Prescription Switching among Rheumatologists: A Saudi Society for Rheumatology Initiative

**DOI:** 10.1016/j.jsps.2021.10.012

**Published:** 2021-12-21

**Authors:** Mohammed A. Omair, Rana Almadany, Maha A. Omair, Hanan Al Rayes, Haya M. Almalag, Aws Alshamsan

**Affiliations:** a Rheumatology Unit, Department of Medicine, King Saud University, Riyadh, Saudi Arabia; b Saudi Food and Drug Authority, Saudi Arabia; c Department of Statistics and Operations Research, College of Science, King Saud University, Riyadh, Saudi Arabia; d Rheumatology Unit, Medicine Department, Prince Sultan Military Medical City, Riyadh, Saudi Arabia; e Clinical Pharmacy Department, College of Pharmacy, King Saud University, Riyadh, Saudi Arabia; f Department of Pharmaceutics, College of Pharmacy, King Saud University, Riyadh, Saudi Arabia

**Keywords:** Biosimilars, Non-medical switching, Rheumatoid arthritis, Saudi Arabia

## Abstract

**Background:**

The aim of this study was to evaluate rheumatologists’ perceptions of biosimilar biologics and Non-Medical Switching (NMS).

**Methods:**

A cross-sectional survey was conducted among registered members of the Saudi Society for Rheumatology. The questionnaire focused on biosimilars and NMS. Logistic regression was performed to ascertain the effect of demographics and practice characteristics on the use of biosimilars and NMS.

**Results:**

Out of 249 SSR members, 143 completed the survey, generating a response rate of 57.4%. Of those (59.44%) were men with a mean (±SD) age and years of practice of 42.3 ± 9.13 and 10.3 ± 8.9, respectively. Rheumatologists managing adult patients (81.82%) and Ministry of Health practice (43.36 %) were the majority of respondents. Previous experience in prescribing a biosimilar was reported by 43 (30.07%) participants, with a higher probability among women (p = 0.015). A total of 26 (18.18%) participants had performed NMS on eligible patients. Adequate knowledge on biosimilars was reported by 69 (48.25%) participants. The adequacy of evidence to grant biosimilar approval for the studied indication and extrapolation to treat other conditions was reported by 88 (61.5%) and 69 (48.3%), respectively. The concept of *totality-of-the-evidence* was well understood by 37.1%. Biosimilars had been previously used by 43 (30.07) participants in their practice. NMS had been attempted by 26 (18.18), while 86 (60.1%) participants believed that NMS might harm patients.

**Conclusion:**

There is a clear knowledge gap about the biosimilar approval process among adult and pediatric rheumatologists who took part in the survey. In addition, a large number of participants reported having negative opinions about NMS. There is a need to organize SSR-led educational activities, and develop national guidelines regarding biosimilars and NMS.

## 1 Introduction

Rheumatic diseases are associated with a significant burden on patients and the community in Saudi Arabia (Omair et al., 2020). Biologics have led to a dramatic improvement in the long-term outcomes of patients with rheumatic diseases; however, this has come with an increase in direct costs to health care systems (Curtis and Singh, 2011). The introduction of biosimilars to the clinical practice of rheumatology has led to significant cost savings and improved access to treatment with biologics in many countries (Baumgart et al., 2019). Different levels of uptake regarding biosimilars have been observed worldwide. This could be related to economic pressures, heterogeneity of regulatory procedures, and differences in strategies on how to introduce biosimilars into health systems (Kim et al., 2020). All of these factors apply to the Middle East region; which faces many healthcare-related challenges, and has been shown to have a varying acceptance of biosimilars (El Zorkany et al., 2018). Due to a lack of national guidelines, the levels of understanding and use of biosimilars in Saudi Arabia are low, and need to be addressed (Halabi et al., 2018). Rheumatologists are important stakeholders who play a critical role in facilitating biosimilar uptake. When switching from a familiar biologic therapy to a biosimilar therapy is undertaken, adequate communication with patients and caregivers, assessment of the continuity of medical care, and verification of the development of adverse events are main support tasks that need to be completed. Previous studies in other countries have uncovered some concerns and issues regarding the use of biosimilars that are primarily the result of an incomplete understanding of biosimilar manufacturing and approval processes (Omair et al., 2020, Omair et al., 2017). The main obstacles to complete understanding that have been reported are as follows: 1) comprehension of the concept, *totality-of-the evidence*; 2) extrapolation to all approved indications based on successful clinical trials in a model disease; and 3) inadequate long-term safety and efficacy data (Alten, 2015).

In Saudi Arabia, obstacles similar to the above have been found, in combination with a lack of educational activities targeted to healthcare providers, and a slower introduction of biosimilars in the field of rheumatology. Additionally, there is a need for stronger and continuous collaborative efforts between healthcare providers and regulatory bodies.

The aim of this survey was to explore perceptions of rheumatologists in Saudi Arabia regarding biosimilars and non-medical switching (NMS).

## 2 Methods

This was a cross-sectional study using a survey that was conducted between November 1st and November 30th, 2020. Registered members of the Saudi Society for Rheumatology (SSR) who were in the database were invited by an official email to respond to the survey. Adult and pediatric rheumatologists, in-training fellows, and service specialists were included.

### 2.1 Questionnaire

The questionnaire was partially adapted from a study published by Narayanan and Nag in 2016 (Narayanan and Nag, 2016), and a study we conducted in a group of Arab rheumatologists, published in 2020 (Omair et al., 2020). It was made up of 4 sections, as follows:

1. Demographics (8 questions).
  • Participants were asked about their gender, age, nationality, professional specialty, years of practice, and current position.
  • They were also asked if they had ever prescribed a biosimilar before,
  • and if they had ever performed non-medical switching.
2. Knowledge of and experience with biosimilars (7 questions).
  • How would you grade the level of your knowledge on biosimilars?
  • Do you believe that the evidence published on biosimilars is adequate to grant approval?
  • Do you believe that the evidence published on biosimilars is adequate to grant extrapolation for treatment of other indications?
  • Do you have a clear understanding of the concept of *the-totality-of-the- evidence* regarding the approval process of biosimilars?
  • What is the likelihood of your prescribing a biosimilar to an eligible patient with rheumatic disease at the current moment?
  • How long should a small group of patients with rheumatic diseases use a biosimilar, before you would feel comfortable prescribing them to a large number of patients?
  • In your opinion, what are the top three factors that are obstacles to large-scale use of biosimilars? (Multiple options allowed).
3. Knowledge and experience about NMS (4 questions)
  • Do you believe that non-medical switching can be harmful to patients?
  • Do you believe that non-medical switching will lead to significant cost savings?
  • Are you willing to perform non-medical switching with your patients when needed?
  • What is the cost difference between the originator biologic and its biosimilar that would be expected, in order to justify non-medical switching?
4. Educational activities related to biosimilars and NMS (2 questions)
  • Do you believe that educational activities related to biosimilars sponsored by a single pharmaceutical company are biased?
  • Do you believe that the Saudi Society for Rheumatology should plan more activities related to biosimilars, non-medical switching, and pharmacoeconomics?

### 2.2 Statistical analysis

Demographic data were summarized as descriptive statistics. Logistic regression was performed to ascertain the effect of demographics and practice characteristics on the use of biosimilars and NMS. A chi-square test of independence was used to test associations between variables. All statistical analyses were performed using the Statistical Package for the Social Sciences software (version 18.0; IBM Corp., Armonk, NY, USA).

### 2.3 Ethical considerations

The study was approved by the Institutional Review Board, IRB, of the College of Medicine, King Saud University (E-). All participants provided signed informed consent prior to study enrolment.

## 3 Results

### 3.1 Demographics

A total of 249 members were registered in the SSR database. The survey was opened by 150 members and 143 completed the questionnaire, generating a response rate of 57.4%. Of these, 85 (59.4%) were men, 113 (79%) were Saudis, 115 (80.42) were consultants, and 117 (81.8%) were rheumatologists treating adults. Saudi rheumatologists were more likely to work in academic centers (30.1% vs. 6.7%, p < 0.0001) and have a pediatric specialty (22.1% vs. 3.3%, p = 0.007). The mean (±SD) age and years of practice were 42.3 (±9.13) and 10.3 (±8.9), respectively Table 1.

**Table 1:** Baseline characteristics of the study participants.

Characteristic	N (%)
Males	85 (59.44)
Saudi nationality	113 (79.02)
Mean age	42.3 ± 9.13
Mean Years of Practice	10.3 ± 8.9
Specialty	
Adult rheumatologist	117 (81.82)
Pediatric rheumatologist	26 (18.18)
Consultant level	115 (80.42)
Type of practice	
Ministry of health	62 (43.36)
Military institution	28 (19.58)
Academic centre	36 (25.17)
Private practice	17 (11.89)
Ever used a biosimilar	43 (30.07)
Performed Non-Medical Switching	26 (18.18)

Participants employed by the Ministry of Health hospital formed the largest proportion of the sample, accounting for 43.4%, followed by academic centers (25.2%), military institutions (19.6%), and the private sector (11.9%). Previous experience with prescribing biosimilars was reported by 43 (30.07%) participants, with a higher probability among women respondents (p = 0.015). A total of 26 participants (18.18%) had performed NMS with eligible patients. All of the above are shown in Table 2

**Table 2:** Variable impact on participants’ responses.

Question: What is the likelihood of prescribing a biosimilar to an eligible rheumatic disease patient at the current moment?			
Variable	Odds ratio	CI	p value
Male gender	1.553	0.728–3.311	0.255
Non-Saudi nationality	1.175	0.426–3.238	0.756
Adult specialty	2.183	0.84–5.677	0.109
Consultant status	1.18	0.473–2.944	0.722
Type of practice			
Academic	1.169	0.499–2.737	0.72
Military	3.901	1.334–11.407	**0.013**
Private practice	2.975	0.754–11.735	0.119
Constant	0.364	–	0.113
Do you have a clear understanding on the concept of totality of evidence regarding the approval process of biosimilars?			
Variable	Odds ratio	CI	p value
Male gender	0.766	0.358–1.637	0.491
Non-Saudi nationality	0.409	0.149–1.127	0.084
Adult specialty	0.571	0.211–1.549	0.271
Consultant status	0.366	0.13–1.031	0.057
Question: Do you believe that non-medical switching could be harmful?			
Variable	Odds ratio	CI	p value
Female gender	1.847	0.86–3.965	0.116
Non-Saudi nationality	1.727	0.616–4.842	0.299
Adult specialty	1.043	0.403–2.699	0.93
Consultant status	1.368	0.544–3.437	0.505
Type of practice			
Academic	0.758	0.326–1.764	0.52
Military	2.386	0.865–6.581	0.093
Private practice	1.55	0.396–6.067	0.529
Constant	0.706	–	0.616
Question: Do you believe that non-medical switching could lead to significant cost-savings?			
Variable	Odds ratio	CI	p value
Female gender	1.418	0.669–3.002	0.362
Saudi nationality	1.093	0.416–2.869	0.857
Pediatric specialty	1.341	0.514–3.5	0.548
Trainee status	1.789	0.679–4.716	0.239
Type of practice			
Academic	0.705	0.282–1.764	0.455
Military	2.232	0.884–5.635	0.089
Private practice	1.4	0.394–4.97	0.603
Constant	0.952	–	0.925

### 3.2 Biosimilars

**Fig. 1:**
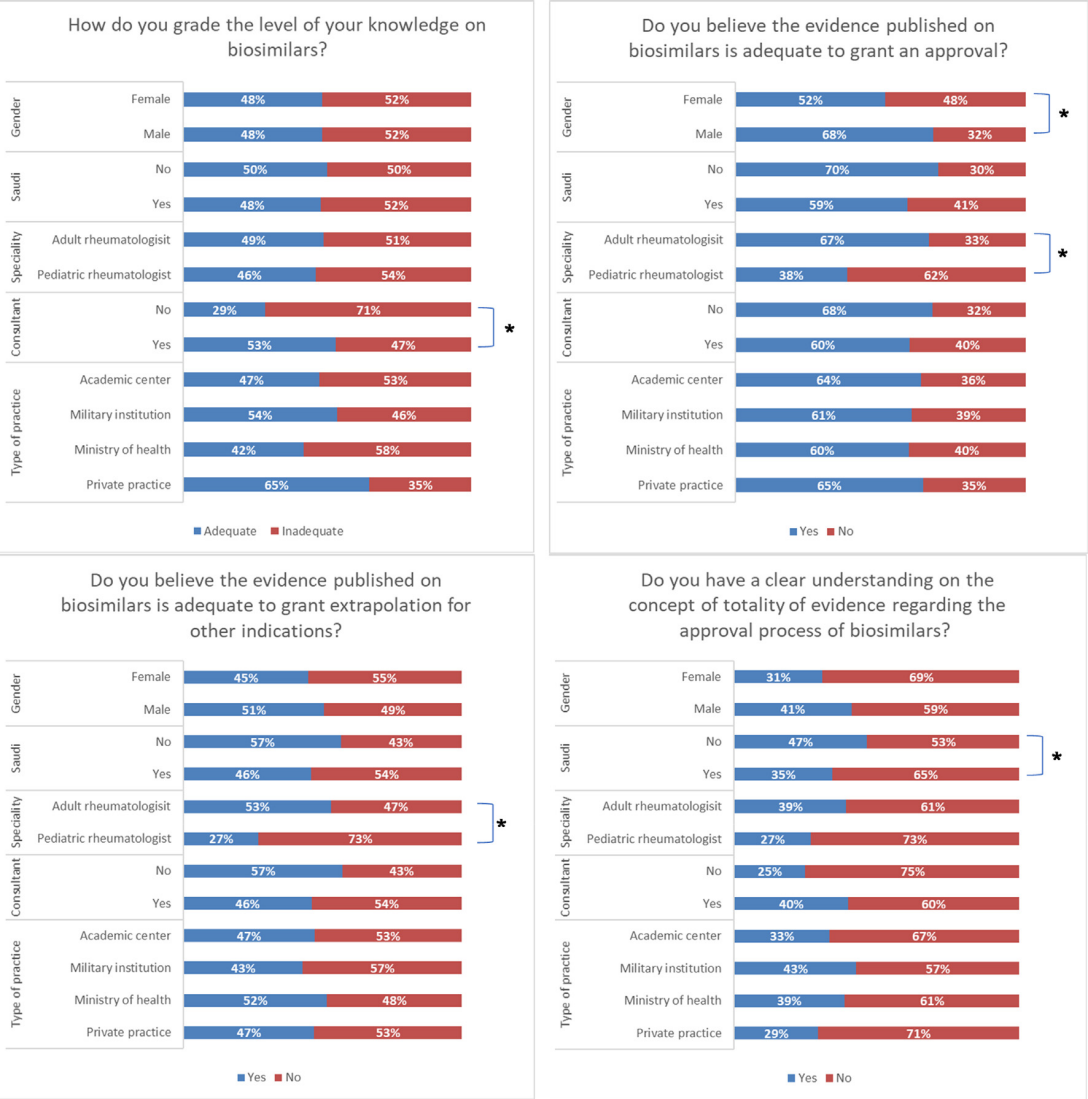
Impact of demographics and practice characteristics on response to questions on biosimilar *p < 0.05.

**Fig. 2:**
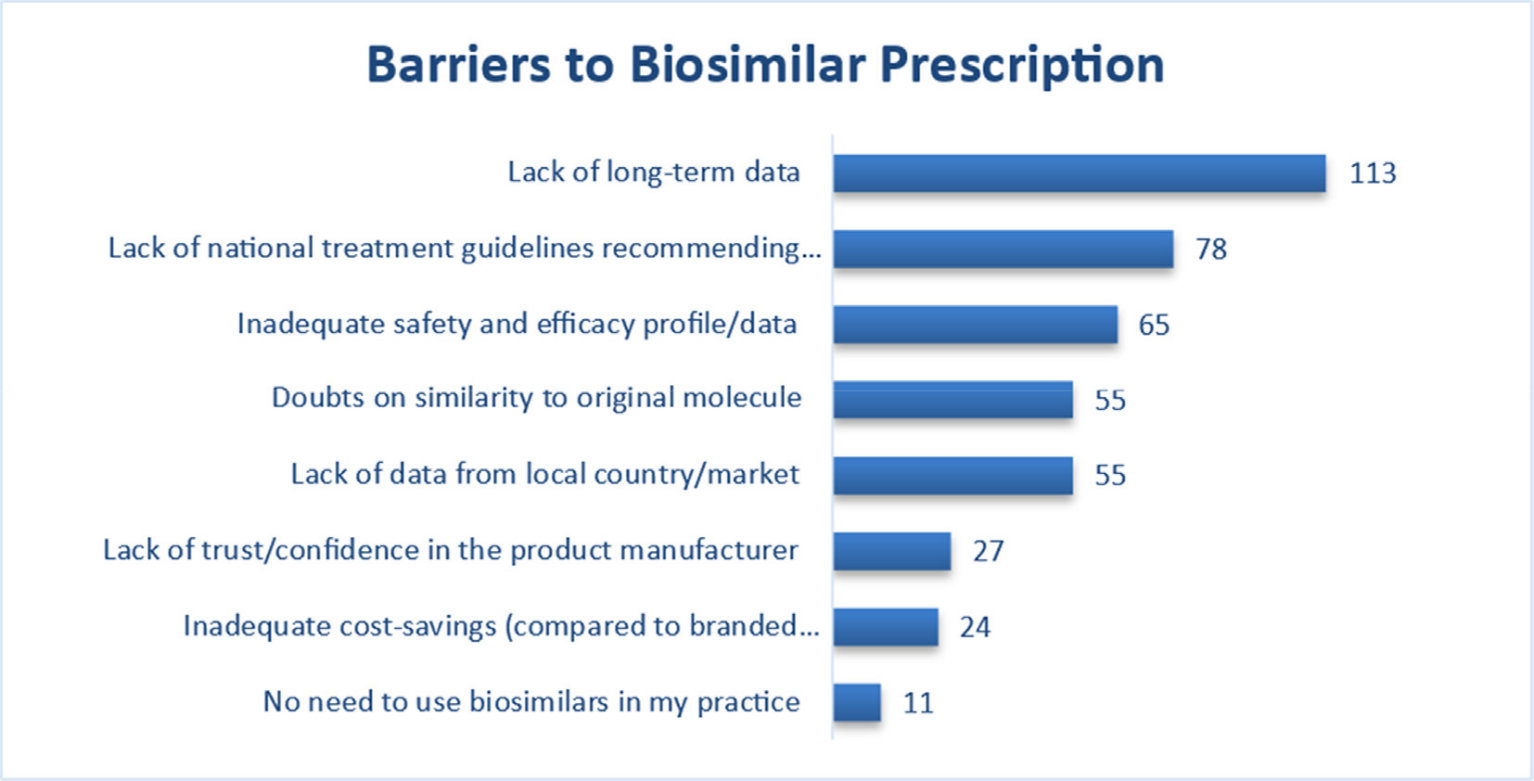
Factors that affect biosimilar prescribing among participants.

An adequate level of knowledge about biosimilars was reported by 69 respondents (48.25%). When asked about the level of evidence needed to grant approval of a biosimilar for a studied indication, 88 of those responding (61.5%) believed it was adequate. Of these, men and rheumatologists managing adult patients were more likely to respond “yes” (68% vs. 32%, p = 0.047) and (66.7% vs. 38.5%, p = 0.008), respectively. In addition, 69 (48.3%) of participants thought that the current published evidence is adequate to allow extrapolation of biosimilars to all indications approved for the originator biologic, with rheumatologists who treat adult patients more likely to respond “yes” (53.0% vs. 26.9%, p = 0.014). The majority of participants (62.9%) reported inadequate understanding of the concept of *the-totality-of-the evidence* that is used to approve biosimilars. Currently, 87 participants (60.8%) answered that they are willing to prescribe a biosimilar to an eligible patient (Fig. 1). However, when asked about their willingness to prescribe biosimilars on a large scale, (58.7%) and (25.2%) were more comfortable if they first build on 1–2- and–3–5-year patient experiences, respectively. Lack of long-term data, absence of national biosimilar guidelines, and inadequate safety/efficacy data were the top three obstacles reported that are currently preventing rheumatologists from using biosimilars (Fig. 2). At the current time, military practitioners were 3.901 times (CI:1.334–11.407) more likely to have prescribed a biosimilar (p = 0.013).

### 3.3 Non-Medical switching

Eighty-one (56.6%) participants were willing to perform NMS with their patients. NMS was believed to be harmful by 86 (60.1%) participants. On the other hand, 88 (61.5%) believe that NMS can lead to significant cost savings to the healthcare system. A mean of 51.48 ± 19.24 % responded that a reduction in the price of biosimilars justifies performing NMS for patients.

### 3.4 Educational activities

Regarding event management, 112 (78.3%) participants believed that educational activities about biosimilars sponsored by a single pharmaceutical company are biased. As a result, 135 (96.5%) participants believed that these activities should instead be organized by SSR. Full analysis was performed on responses of consultant participants only, with no significant impact on the results (data not shown).

### 3.5 Biosimilar and Non-Medical switching experience

Among those who reported the prior use of biosimilars, 35 (81.4%) were in governmental hospital through a NMS procedure or biosimilar prescription due to automatic subsititution in the hospital formulary. While the remaining prescribed biosimilars in the private practice through a charity organization. When asked about their perception on their perceived knowledge on biosimilars, evidence on published evidence, extrapolation and the concept of totality-of-evidence 8 (18.6%), 9 (20.9%), 18 (41.9%) and 20 (46.5%) reported it to be inadequate. Also 29 (67.4%) and 23 (53.5%) of these participants believe that NMS can be harmful and will not lead to significant cost-savings.

## 4 Discussion

This survey is an important initiative that provides information to guide the SSR in designing educational activities and disseminating unbiased information about biosimilars. Prior to the approval of biosimilars in Saudi Arabia in 2015, we conducted a survey on rheumatoid arthritis (RA) treatment strategies. Of the 54 participants in that study, 26.3% used biosimilars prior to originators (Omair et al., 2017), whereas in the current study, we showed that about 60% of respondents indicated they are currently ready to start using biosimilars for treatment. We also conducted a study on Arab rheumatologists during a regional meeting held in 2018 and found that only 30% of participants expressed that they were either definitely or highly likely to prescribe a biosimilar to an eligible patient.

The timing of this study, during the COVID-19 pandemic, is associated with increased economic pressure on the healthcare system and a shortage of medications. This might impacted the responses of participants. There is currently an increasing demand for biosimilars, especially with the economic impact of COVID-19 that has affected the medical supply chain across the world. This has led to drug shortages, which in turn may mandate the development of guidelines on the use of biosimilars and NMS.

A systematic review by Odinet et al. showed that higher discontinuation rates of biosimilars resulting from adverse events reported in open-label studies, compared to that observed in blinded randomized controlled trials, which could be attributed to the nocebo effect (Odinet et al., 2018). An inadequate understanding of the differences in the biosynthesis of biosimilars compared to their originators and the reversed ratio of preclinical/clinical regulatory requirements are two important shortcomings that impacted the perception of the rheumatologists responding to our survey. This might have led to unacceptance and an increased risk of the nocebo effect (Barsky et al., 2002). We found that a large proportion of the study participants reported inadequate knowledge about biosimilars and their manufacturing processes. Similar findings were reported from a study conducted by Chapman et al. that included consultants and specialists registered in nine medical societies/associations in the United Kingdom. Rheumatologists formed 26% of that sample. The survey revealed that 18% of participants thought that biosimilars were generic drugs, and 6% did not know in what areas of medicine biosimilars are used, or never heard this term before. In a different study, a subgroup analysis based on subspecialty found that more than half of rheumatology consultants expressed minor to major concerns regarding the efficacy or safety of biosimilars (Chapman et al., 2017). Another US study published by Cohen et al. showed that only half of 1201 clinicians from medical specialties with high biologicals use understood the concept of the*-totality-of-the- evidence*. In addition, a subgroup analysis that included rheumatologists, found that 58.5% and 56% believe that biosimilars are comparable to their originators in terms of efficacy and safety, respectively (Cohen et al., 2016). Beck et al. conducted a French study of 116 rheumatologists. Of those, 40.6% rated their overall knowledge of biosimilars as either good or very good. When asked about biosimilars’ quality, safety, and efficacy, 50%, 44%, and 54.3% agreed to be adequate (Beck et al., 2016).

A systematic review published by Halimi et al. in 2020 evaluated 16 qualitative and quantitative methodologies. The studies included a variety of healthcare providers as participants. The authors observed variability in knowledge and understanding of biosimilars (Halimi et al., 2020).

In our survey, we found that pediatric rheumatologists were more likely to question the published evidence, and not agree with the extrapolation process. This could be due to the fact that the trials generally target a sensitive population, which in the case of rheumatology would be RA or spondylarthritis, and often exclude vulnerable populations, such as children and patients with comorbidities. However, the FDA in the United States and the European Medicine Agency have not required additional studies focused on children and young people (Aragon Cuevas and Hedrich, 2020). This has led individual centers to develop committees to evaluate the needs and challenges of adopting biosimilars in children (Aragon Cuevas and Hedrich, 2020). Most of the current real-world evidence on prescribing biosimilars or performing NMS has been published as abstracts or is pending publication. Most pediatric rheumatologists practicing in Saudi Arabia are working in tertiary care centers. We did not identify differences in knowledge and acceptance in this group, compared with those practicing in adult specialties.

NMS between originators and their biosimilar products is a complex issue. Clinical trials of a single switch have been reassuring in the case of infliximab for treatment of both RA and Inflammatory Bowel Diseases (IBD) (Feagan et al., 2019). These results provided the background for a nationwide switch in various countries, such as Norway (Glintborg et al., 2017) But other societies have issued more conservative statements, for example, Italy (Atzeni et al., 2015). Although strong evidence is currently lacking, the use of biosimilars could help support the management of rheumatic diseases under the current economic burden of COVID-19 (Yoo, 2014).

In the current study a large proportion of participants who previously prescribed biosimilars reported inadequate knowledge on biosimilars and their evidence. Additionally, most of them believe that NMS can be harmful and will not lead to significant cost-savings.

In the final part of our survey, we observed that respondents believe that biosimilar-related educational activities are subject to bias, and that respondents believe that these activities need to be organized by SSR.

We previously published a white paper describing a symposium that was presented in collaboration with three scientific societies (the SSR, the Saudi Pharmaceutical Society, and the Saudi Society of Clinical Pharmacy) on biosimilars. The white paper identified that the development of national guidelines on biosimilars use was the unmet need with the highest priority (Omair et al., 2020). This is consistent with the barriers that were identified in our study.

Limitations of our study include the use of a non-validated questionnaire; under-representation of participation from some health sectors, such as the private sector; and a small number of respondents with previous exposure to biosimilars and NMS. All of these could have impacted the responses. We also did not explore the effect of the type of biosimilar or its producing company on perception.

In conclusion, this initiative has identified critical knowledge gaps regarding the biosimilar approval process and other barriers that likely affect their use by rheumatologists in Saudi Arabia. It also identified negative views on NMS, which should be the target of future educational activities.

## Funding

This study was supported by an unrestricted grant from SSR.

## Declaration of Competing Interest

Mohammed A. Omair has received speaker’s honorarium from Abbvie, Actelion, Amgen, Brystol Myers Squibb, Glaxo-Smith-Kline, Hekma, Janssen, New Bridge, Novartis, Pfizer, and Roche. Hanan Al Rayes has received speaker’s honorarium from Abbvie, Amgen, Glaxo-Smith-Kline, Janssen, New Bridge, Novartis, and PfizerAws Alshamsan has received speaker’s honorarium from Amgen, Sandoz, Abbvie, and Eli Lilly. Maha A. Omair, Rana Almadany and Haya M. Almalag disclose no conflict of interest.
